# eSport consumption and sponsor brand equity: the mediating role of congruence

**DOI:** 10.3389/fpsyg.2025.1594068

**Published:** 2025-07-03

**Authors:** Yeayoung Noh, Yoonji Ryu, Kyungyeol Kim

**Affiliations:** ^1^Department of Physical Education, Seoul National University, Seoul, Republic of Korea; ^2^Division of Global Sport Industry, Hankuk University of Foreign Studies, Seoul, Republic of Korea; ^3^Department of Life Sport Education, Kongju National University, Gongju, Republic of Korea

**Keywords:** eSport industry, live attendance, live streaming, congruence, brand equity

## Abstract

The present study examines how eSport consumption influences sponsor brand equity through the mediating role of sponsor-eSport congruence. Specifically, the current study investigates the mediating effects of congruence in the relationship between two types of eSport consumption—live attendance and live streaming—and three types of sponsor brand equity: brand loyalty, brand image, and brand awareness. Data were collected from 1,353 Korean eSport fans. The findings reveal that live attendance positively affects brand image but has no significant effect on brand loyalty or brand awareness. Similarly, live streaming enhances brand image but does not influence brand loyalty or brand awareness. Mediation analyses indicate that congruence fully mediates the relationship between live attendance and both brand loyalty and brand awareness, while partially mediating the relationship between live attendance and brand image. Congruence fully mediates the relationship between live streaming and both brand loyalty and brand awareness, while partially mediating the link between live streaming and brand image. These results indicate the critical role of congruence in translating eSport consumption into stronger sponsor brand equity, offering valuable insights for both researchers and practitioners navigating the rapidly growing eSport sponsorship landscape.

## 1 Introduction

eSport has emerged as one of the most rapidly expanding global professional sport industries, captivating millions of viewers and transforming the landscape of entertainment and competitive gaming (Abdolmaleki et al., 2025; Kim et al., 2025). With major tournaments attracting audiences comparable to traditional sporting events, the eSport industry has evolved into a multi-billion-dollar market, fostering a complex ecosystem of players, organizations, media platforms, and sponsors (Pizzo et al., 2022). As the eSport industry continues to grow at an unprecedented pace, scholarly interest has also intensified, leading to a surge of academic inquiry across multiple disciplines (Flegr and Schmidt, 2022; Pizzo et al., 2022). Despite this increasing scholarly attention, the existing literature remains fragmented, focusing primarily on three key areas: defining the concept of eSport, analyzing the cognitive and behavioral characteristics of eSport consumers, and investigating the social and physiological impacts of eSport participation (Flegr and Schmidt, 2022). While these areas provide valuable insights into the phenomenon of eSport, there remains a critical gap in understanding the role of sponsorship within this domain. This omission is particularly significant given that sponsorship is the largest revenue driver for the eSport industry, accounting for over 60% of total market revenue (Newzoo, 2022). Therefore, a comprehensive examination of eSport sponsorship and its influence on consumer perceptions is essential for both academics and industry stakeholders.

Sponsorship in eSport presents a unique opportunity for brands to engage with a highly lucrative, digitally engaged, and predominantly young audience segment (Huettermann et al., 2023). This demographic is particularly attractive to marketers due to its purchasing power, technological savviness, and resistance to traditional advertising channels (Meng-Lewis et al., 2024). Recognizing the commercial potential of eSport, major corporations—such as Coca-Cola, Intel, Comcast, and Audi—have made substantial investments in sponsoring eSport leagues, tournaments, and teams (Rogers et al., 2020). These sponsorships serve as strategic marketing tools designed to enhance brand equity by influencing its core dimensions: brand image, brand loyalty, and brand awareness (Brand and Ströbel, 2024; Mazodier and Merunka, 2012). Brand equity, broadly defined as the value consumers attribute to a brand, represents a crucial intangible asset that drives consumer preference, fosters brand loyalty, and enhances long-term profitability (Keller, 1993). Consequently, understanding how eSport sponsorship contributes to building and sustaining brand equity is vital for organizations aiming to optimize the effectiveness of their sponsorship investments and maintain competitive advantage in an increasingly digital marketplace.

In sponsorship research, congruence has been recognized as one of the most critical factors to consider (Woisetschläger and Michaelis, 2012). Congruence reflects the degree to which consumers perceive a logical and meaningful association between a brand and the sponsored activity (Speed and Thompson, 2000). Extensive research consistently demonstrates that higher levels of congruence positively influence key consumer outcomes, including sponsor recognition, brand favorability, and purchase intention (Hutabarat and Gayatri, 2014; Woisetschläger and Michaelis, 2012). Despite the well-established impact of congruence in traditional sport contexts, its underlying mechanisms and outcomes within the eSport ecosystem remain underexplored. Given the distinct characteristics of eSport—such as its digital-first environment, global reach, and interactive fan engagement—there is a need to examine whether and how congruence operates in this rapidly evolving domain. As eSport sponsorship continues to grow as a primary marketing strategy, understanding the role of congruence within this unique context is essential for optimizing sponsorship effectiveness and informing strategic decision-making.

In light of these gaps, the present study examines how eSport consumption influences brand equity through the mediating roles of sponsor-eSport congruence. Specifically, the present study investigates the mediating effects of congruence in the relationship between eSport consumption (i.e., live attendance and live streaming) and sponsor brand equity (i.e., brand loyalty, brand image, and brand awareness). By doing so, the present study seeks to offer insights for eSport sponsors, helping them optimize sponsorship strategies and enhance brand equity. Ultimately, the findings aim to inform practitioners and advance scholarly understanding of the dynamics of sponsorship within the rapidly evolving eSport landscape.

## 2 Literature review

### 2.1 eSport sponsorship

Sponsorship is commonly understood as “an investment, in cash or in-kind, in an activity, in return for access to the exploitable commercial potential associated with that property” (Meenaghan, 1991, p. 36). Sponsorship represents a critical element of strategic marketing, offering companies a platform to enhance brand visibility, strengthen consumer relationships, and create meaningful brand associations (Cornwell et al., 2024). In the realm of sport, sponsorship has demonstrated its ability to deliver tangible value, serving as building brand equity and achieving marketing objectives (Cornwell and Kwon, 2020). Specifically, sponsorship enables companies to tap into the emotional and psychological bonds that consumers form with the sponsored property, fostering long-term loyalty and favorable brand perceptions (Hutabarat and Gayatri, 2014).

In the context of eSport, sponsorship has emerged as the dominant revenue stream, contributing to approximately 60% of the industry’s total revenue in 2022 (Newzoo, 2022). Unlike traditional sport, the digital-first nature of eSport allows brands to engage with a highly engaged, tech-savvy, and predominantly younger audience that is difficult to reach through conventional advertising channels (Huettermann et al., 2023). As a result, eSport sponsorship offers a distinctive opportunity for companies to cultivate meaningful connections with this demographic by aligning their brands with the passion, excitement, and innovation inherent in the eSport culture. This dynamic environment allows sponsors to leverage immersive digital experiences and interactive platforms to strengthen brand equity and foster long-term consumer relationships.

Prominent examples highlight the growing strategic importance of eSport sponsorships. Mastercard, for instance, has been a key player in esports marketing initiatives, particularly through its exclusive partnership with League of Legends (LoL). Since 2018, Mastercard has leveraged its sponsorship to engage with fans across more than 10 countries through various activations, including experiential events, branded content, and promotional campaigns tied to LoL’s global tournaments. Similarly, Korean Air entered the eSport sponsorship space by establishing an official uniform partnership with South Korea’s eSport national team.

Corporate investment in eSport also extends to partnerships with professional teams. For example, Mercedes-Benz Korea has demonstrated a creative approach by sponsoring the League of Legends Champions Korea (LCK) team. Through this collaboration, Mercedes-Benz Korea provided electric vehicles to high-profile players, such as Faker Sang-hyuk Lee, one of the most celebrated eSport players globally. This initiative reflects the brand’s strategy to align its innovative and technologically advanced image with the eSport consumers, reinforcing its appeal to a consumer base that values cutting-edge technologies and progressive values. These examples indicate the versatility and potential of eSport sponsorship to deliver impactful results. By aligning with eSport properties, companies not only gain access to a growing global audience but also establish themselves as integral participants in a rapidly evolving cultural phenomenon.

### 2.2 eSport consumption

We conceptualize eSport consumption as comprising live attendance and live streaming of an eSport game. Live attendance and live streaming mirror patterns observed in traditional sport consumption, where live attendance reflects physical and social consumption, while live streaming represents technologically mediated and often more individualized consumption (Sjöblom et al., 2020). Both live attendance and live streaming represent unique consumer behaviors that differ in experience, context, and psychological engagement, yet collectively contributing to a comprehensive understanding of eSport consumption (Sjöblom et al., 2020).

The integration of these two consumption forms is particularly relevant in the eSport context. Live attendance provides a tangible and immersive experience, allowing fans to gather at physical venues to support their favorite teams, experience the intensity of competition, and connect with like-minded spectators in a shared social environment (Pu et al., 2022). In contrast, live streaming offers a digital, highly accessible platform that caters to a global audience, enabling personalized and interactive experiences. As the eSport industry continues to evolve alongside advancements in digital technology, live streaming has emerged as a critical medium for reaching a digitally savvy fan base. eSport leagues and organizations have embraced this shift by developing streaming platforms or partnering with digital powerhouses such as Twitter and Amazon to broadcast events, expanding their reach and adapting to the demands of a global audience (Qian and Seifried, 2023). Live attendance and live streaming, therefore, represent distinct yet complementary forms of eSport consumption (Sjöblom et al., 2020).

### 2.3 The effects of eSport consumption on sponsor brand equity

Attending live eSport events creates an immersive experience that strengthens emotional bonds with the event, players, and associated sponsors. Social identity theory (Tajfel and Turner, 1979) suggests that individuals derive a sense of belonging by identifying with a group, such as the eSport fan community. Through live attendance, spectators share moments of excitement and emotional highs with like-minded fans, developing their loyalty toward the event, teams, and sponsors (Kim et al., 2019). This loyalty extends to the sponsoring brands, as spectators perceive sponsors as facilitator of their emotional experiences, thereby fostering brand loyalty toward sponsors (Close Scheinbaum et al., 2019).

Venue-based eSport spectating can also enhance brand image by associating sponsoring brands with positive and memorable fan experiences. According to associative network theory (Anderson, 1983), the positive emotions generated from live spectating become linked to the sponsoring brand in consumers’ memory. For example, Mercedes-Benz’s sponsorship of eSport events conveys a progressive and innovative brand image, resonating with a young, tech-savvy audience (Boronczyk and Breuer, 2021). Consequently, live spectating offers sponsors the opportunity to cultivate a favorable brand image.

Live spectating can boost brand awareness due to the high visibility of sponsor logos, advertisements, and on-site activations at event venues. Sponsors leverage live events to engage audiences through activities such as product sampling or interactive booths, which leave lasting impressions on consumers (Cornwell and Kwon, 2020). Thus, exposure to brands within an exciting and emotionally charged environment, such as a live eSport event, enhances the likelihood of brand recall and recognition.

*H1*: Live attendance positively influences brand loyalty (H1a), brand image (H1b), and brand awareness (H1c).

Live streaming of eSport events through platforms such as Twitch and YouTube offers a personalized and interactive consumption experience that strengthens brand loyalty. By engaging directly with content, viewers from emotional connections with the events, players, and sponsoring brands. These connections can transfer to sponsoring brands as viewers associate them with the eSport content they enjoy (Teal et al., 2020). Additionally, the convenience and accessibility of live streaming in the home-like environment encourage habitual and frequent consumption, creating repeated exposure to sponsor branding and reinforcing brand loyalty over time (Sjöblom et al., 2020). This habitual engagement strengthens the bond between the viewer and the sponsor, cultivating loyalty through consistent brand presence.

The interactive nature of live streaming can also shape a positive brand image. Sponsors that seamlessly integrate their branding into eSport streams or support community-driven initiatives (e.g., giveaways, promotions, fan engagement) are perceived as authentic and supportive of the eSport audience (Wohn and Freeman, 2020). For example, Red Bull’s integration into eSport streams emphasizes its association with energy, performance, and innovation, aligning with eSport culture. This alignment builds favorable associations with the sponsoring brand, reinforcing a strong and positive brand image. The two-way interaction inherent in live streaming creates an immersive and engaging experience, making sponsoring brands appear relevant and culturally attuned to the eSport community (Wohn and Freeman, 2020).

Live streaming drives brand awareness through persistent brand visibility across digital platforms. Streamed content often features sponsor logos, integrated advertisements, and verbal mentions by players or casters, ensuring consistent exposure throughout the viewing experience (Sjöblom et al., 2020). According to the mere exposure effect (Zajonc, 1968), repeated exposure to sponsor branding increases familiarity and recognition among viewers. This effect is particularly pronounced in eSport, where audiences often watch streams for extended periods, sometimes exceeding several hours per session (Wohn and Freeman, 2020). The prolonged exposure during these sessions embeds sponsoring brands into viewers’ memory, amplifying brand recall and recognition (Davtyan and Tashchian, 2022).

*H2*: Live streaming positively influences brand loyalty (H2a), brand image (H2b), and brand awareness (H2c).

### 2.4 The mediating effects of congruence

Attending live eSport events provides spectators with immersive, multi-sensory experiences that foster stronger associations with the event, participating teams, and sponsoring brands (Jang et al., 2020; Kim et al., 2019). This immersive environment allows spectators to engage with the event through direct emotional and social experiences, such as cheering with fellow fans, interacting with players, and participating in branded activities (Kim et al., 2019). During these moments, sponsors become closely associated with the excitement and positive emotions of the event through visible branding elements, including logos, interactive booths, and on-site promotions (Boronczyk and Breuer, 2021). This consistent and meaningful presence can enhance the perception that the sponsor aligns naturally with the eSport environment, reinforcing congruence in consumers’ minds. Additionally, the sustained exposure to sponsor messages throughout the event—whether on digital screens, player uniforms, or venue installations—reinforces the perceived fit between the sponsor and the eSport setting (Cornwell and Kwon, 2020). Over time, these repeated exposures help solidify the connection between the sponsor and the positive experiences fans associate with eSport events (Zajonc, 1968).

Live streaming of eSport events through digital platforms such as Twitch and YouTube can also enhance congruence through personalized and interactive experiences. Unlike live attendance, live streaming allows spectators to engage remotely while participating in real-time discussions via live chat and social media (Qian and Seifried, 2023). This dynamic environment enables sponsors to interact directly with viewers through branded content, giveaways, and other interactive features, making their presence feel more integrated and relevant to the eSport experience (Sjöblom et al., 2020). Furthermore, live streaming offers extended and repeated exposure to sponsor branding. Throughout the broadcast, sponsor logos, advertisements, and verbal mentions by commentators are consistently presented, increasing familiarity with the brand (Qian and Seifried, 2023). As viewers spend hours engaging with eSport content, the prolonged exposure reinforces the perception that sponsors are an integral part of the eSport ecosystem (Davtyan and Tashchian, 2022). This alignment between the sponsor and the eSport context enhances the perceived congruence, which in turn strengthens the brand’s relevance and authenticity within the community.

The perceived congruence between eSport and sponsoring companies plays a crucial role in enhancing brand loyalty. When consumers perceive a strong alignment between the sponsor and the sponsored entity, they are more likely to infer shared values and mutual goals between the two (Keller, 1993). This alignment fosters emotional connections and strengthens consumers’ identification with the brand, as proposed by social identity theory (Tajfel and Turner, 1979). For example, when a tech-savvy sponsor such as Intel supports an eSport team, fans perceive the sponsor as inherently compatible with the eSport ecosystem. This congruence cultivates trust and loyalty toward the sponsor, as consumers view the sponsorship as authentic and meaningful (Speed and Thompson, 2000). Moreover, the reduced skepticism about the sponsor’s motives promotes long-term loyalty, as consumers perceive the sponsor’s investment as beneficial to their community and interests (Moharana et al., 2023).

Perceived congruence also positively influences brand image by creating favorable associations in the minds of consumers. The associative network theory (Anderson, 1983) indicates that the positive attributes of the sponsored entity—such as excitement, competitiveness, and innovation in eSport—transfer to the sponsoring brand when consumers perceive a good match between the two. This process strengthens the brand’s positioning within a specific context, such as technological advancement or youth culture (Speed and Thompson, 2000). Conversely, a lack of congruence may lead to cognitive dissonance, wherein the sponsorship appears forced or inauthentic, negatively affecting the brand image. For example, if a luxury fashion brand sponsors an eSport event without a clear connection to the gaming audience, consumers may question the motives behind the sponsorship, leading to skepticism (Ruth and Simonin, 2003).

Congruence also enhances brand awareness by creating a seamless association between the sponsor and the sponsored entity. Schema theory (Rumelhart, 1980) suggests that consumers process congruent information more efficiently, as it aligns with their pre-existing cognitive structures. When the attributes of the sponsoring company align closely with the values and characteristics of eSport, consumers are more likely to remember the sponsor due to the logical connection between the two. For instance, a brand such as Logitech sponsoring an eSport team directly resonates with the audience’s understanding of gaming peripherals, enhancing the brand’s recall and recognition (Moharana et al., 2023). Additionally, congruence increases the likelihood that sponsor messages will be reinforced during repeated exposures, further solidifying brand awareness (Cornwell and Kwon, 2020).

In sum, congruence serves as a central cue that enhances consumers’ cognitive processing of sponsor-related information, supporting its mediating role. Live attendance and live streaming serve as key platforms for brand exposure, and it is congruence between the sponsor and the eSport entity that ultimately determines the extent to which consumers effectively process and internalize sponsorship messages (Rogers et al., 2020). High congruence ensures that sponsorships resonate with consumers, enhancing the overall impact of brand equity (Aguiló-Lemoine et al., 2020; Cornwell et al., 2024).

*H3*: Congruence mediates the relationship between eSport consumption and brand equity, specifically between live attendance and brand loyalty (H3a), live attendance and brand image (H3b), live attendance and brand awareness (H3c), live streaming and brand loyalty (H3d), live streaming and brand image (H3e), and live streaming and brand awareness (H3f).

## 3 Method

### 3.1 Research context

South Korea was selected as the research context due to its prominent role in the global eSport landscape. Widely recognized as the birthplace of eSport, South Korea has emerged as a major player in the rapidly growing eSport industry (Kim et al., 2025; Pizzo et al., 2022). For example, in 2021, South Korea ranked forth among the world’s top 10 gaming markets, following China, the United States, and Japan (Buchholz, 2021). This ranking is projected to persist through 2025, which is particularly notable given the country’s relatively small population size compared to other leading markets (Buchholz, 2021).

We focus on LoL as the specific eSport game. Within eSport, LoL is known to be a globally popular and highly competitive game. South Korea hosts the League of Legends Champions Korea (LCK), one of the most prestigious professional leagues, alongside the secondary-tier LoL Challengers League. LoL is also home to internationally recognized figures, such as Sanghyeok “Faker” Lee from the T1 team, who is regarded as a cultural icon within the global eSport community. Given LoL’s widespread popularity and competitive environment, the current study focuses on LoL teams participating in the LCK and LoL Challengers League, providing a rich and relevant context for examining the effects of eSport consumption on brand equity.

### 3.2 Data collection and procedure

Data were collected through a Korean market research company, with a specific focus on targeting eSport fans. At the beginning of the online survey, participants were asked to select one team they supported from a list, including teams that were participants in the LCK and LoL Challengers League during the period of survey administration: SKT T1, Samsung Galaxy, LONGZHU Gaming, kt Rolster, ROX Tigers, bbq Olivers, MVP, CJ Entus, and Kongdoo Monster. Participants were then instructed to answer questions about live attendance and live streaming based on their chosen team. Additionally, they were asked to respond to items assessing congruence and brand equity, referring to the main sponsor associated with their selected team.

A total of 1,384 survey responses were initially collected. Following data screening to remove incomplete responses, 1,353 responses were deemed valid for further analysis. Participant demographic details are presented in Table 1. The demographic profile of the participants in the present study aligns with the typical characteristics of eSport consumers (Marcus, 2023).

**Table 1:** Demographic information.

Demographic variable	Frequency	Percentage
Gender		
Male	835	61.71%
Female	518	38.29%
Age		
10s	411	30.38%
20s	790	58.39%
30s	145	10.71%
40s	7	0.52%
Education		
Middle school graduate	272	20.10%
High school graduate	631	46.64%
Bachelor’s degree	409	30.23%
Master’s degree	41	3.03%
Income (per month)		
Less than $1,000	893	66.00%
$1,001-3,000	384	28.38%
$3,001-5,000	58	4.29%
Greater than $5,001	18	1.33%

### 3.3 Measures

Live attendance and live streaming of eSport games were each measured using three items adapted from Sjöblom et al. (2020) on a seven-point semantic differential scale. Sponsor-event congruence was assessed using three items adapted from Bridges et al. (2000), measured on a seven-point Likert scale. To evaluate brand equity, three items each were used to measure brand awareness (Yoo and Donthu, 2001), brand image (Sasmita and Suki, 2015), and brand loyalty (Yoo and Donthu, 2001), all based on a seven-point Likert scale.

Team identification was included as a control variable for brand equity, as prior research indicates that stronger team identification can influence eSport consumers’ behavioral responses toward sponsors (Calapez et al., 2024). Four items adapted from Mael and Ashforth (1992) were used to measure team identification, using a seven-point Likert scale.

The survey instrument was initially developed in English and subsequently translated into Korean by a bilingual researcher fluent in both languages. To ensure linguistic and conceptual equivalence, a second bilingual researcher conducted a back-translation into English. The original English version and the back-translated survey were compared to identify and resolve any discrepancies (Brislin, 1970). This process ensured that the translated survey maintained consistency and validity across both languages.

## 4 Results

### 4.1 Descriptive statistics

Table 2 presents means, standard deviation, and correlations of variables. Mean values ranged from 5.95 (live streaming) to 3.11 (live attendance). All correlations among the variables were positive and statistically significant.

**Table 2:** Means, standard deviation, and correlations.

Variable	1	2	3	4	5	6
1. Live attendance	–					
2. Live streaming	0.25*	–				
3. Congruence	0.15*	0.22*	–			
4. Brand awareness	0.17*	0.24*	0.63*	–		
5. Brand image	0.22*	0.24*	0.72*	0.69*	–	
6. Brand loyalty	0.15*	0.21*	0.70*	0.58*	0.77*	–
Mean	3.11	5.95	4.91	5.48	4.54	4.41
Standard deviation	2.47	1.44	1.84	1.56	1.85	1.91

*p < 0.001.

### 4.2 Measurement model

Confirmatory factor analysis was conducted to test evaluate measurement model. The result indicated an acceptable fit to the data (*χ*^2^ = 857.74, *df* = 48, CFI = 0.95, TLI = 0.94, and RMSEA = 0.06) (Hair et al., 2010). Cronbach’s alpha values for all variables (ranging from 0.80 to. 97) exceeded the recommend threshold of 0.70, thus demonstrating internal consistency (Nunnally and Bernstein, 1994). Composite reliability values ranged from 0.86 to 0.97, surpassing the 0.70 criterion, further supporting the constructs’ reliability (Fornell and Larcker, 1981). Convergent validity was ensured as the average variance extracted (AVE) for all constructs ranged from 0.65 to 0.92, exceeding the recommended cutoff of 0.50 (Fornell and Larcker, 1981). All AVE values were greater than the squared correlations between constructs, providing evidence of discriminant validity (Fornell and Larcker, 1981). Table 3 reports the factor loadings, composite reliability, and AVE values.

**Table 3:** Factor loading, composite reliability (CR), and average variance extracted (AVE).

Items	Factor loading	AVE	CR	α
Live attendance		0.92	0.97	0.95
I go to the stadium to watch [Team Name]‘s game.				
1. Rarely – Always	0.96			
2. Hardly ever – Every game	0.96			
3. Never – Every time	0.95			
Live streaming		0.73	0.89	0.84
I live stream [Team Name]‘s game.				
1. Rarely – Always	0.81			
2. Hardly ever – Every game	0.85			
3. Never – Every time	0.90			
Congruence		0.79	0.92	0.92
I can understand the relationship between ○○○ company and the team.	0.85			
The image of ○○○ company is similar to that of the team.	0.92			
○○○ company and the team have a similar image.	0.91			
Brand awareness		0.67	0.86	0.80
I can recognize ○○○ company among other competing brands.	0.81			
I am aware of ○○○ company.	0.76			
○○○ company is more familiar to me than companies sponsoring other teams.	0.88			
Brand image		0.81	0.93	0.90
○○○ company has a differentiated image in comparison with the other brand.	0.89			
○○○ company has a clean image.	0.93			
○○○ company is well established.	0.88			
Brand loyalty		0.91	0.97	0.97
I consider myself to be loyal to ○○○ company.	0.96			
○○○ company would be my first choice.	0.96			
I will not buy other brands if ○○○ company is available at the store.	0.94			

AVE = Average variance extracted. CR = Composite reliability. α = Cronbach’s alpha.

### 4.3 Structural model

Structural equation modeling was performed to test the hypotheses. The hypothesized model demonstrated a satisfactory fit to the data (*χ*^2^ = 857.74, *df* = 48, CFI = 0.95, TLI = 0.94, and RMSEA = 0.06) (Hair et al., 2010). The results revealed that live attendance positively affected brand image (*β* = 0.06, *p* < 0.01) but did not significantly influence brand loyalty or brand awareness, supporting H1b while rejecting H1a and H1c. Similarly, live streaming positively affected brand image (*β* = 0.06, *p* < 0.01) but showed no significant impact on brand loyalty or brand awareness, supporting H2b while rejecting H2a and H2c. Team identification as the control variable significantly influenced brand loyalty (*β* = 0.17, *p* < 0.001), brand image (*β* = 0.24, *p* < 0.001), and brand awareness (*β* = 0.23, *p* < 0.001).

To test indirect effects, bootstrapping with 5,000 resamples and a bias-corrected 95% confidence interval (CI) was performed. The results indicated the significant full mediating effects of congruence between live attendance and brand loyalty (*β* = 0.08, CI = 0.04 to 0.11), between live attendance and brand awareness (*β* = 0.04, CI = 0.02 to 0.06), while partial mediating effects was found between live attendance and brand image (*β* = 0.07, CI = 0.03 to 0.11). Similarly, the full mediation effects were observed in the relationships between live streaming and brand loyalty (*β* = 0.14, CI = 0.09 to 0.22) and between live streaming and brand awareness (*β* = 0.08, CI = 0.05 to 0.13), while the partial mediation effect was found between live streaming and brand image (*β* = 0.13, CI = 0.08 to 0.21). These results supported the hypotheses H3a through H3f (see the direct and indirect effects in Table 4). The structural model accounted for 57% of the variance in brand loyalty, 60% in brand image, and 38% in brand awareness.

**Table 4:** Direct and indirect effects.

Direct and indirect effects		*β*	*SE*	*p*
Direct effect				
H1a	Live attendance → Brand loyalty	0.01	0.02	0.70
H1b	Live attendance → Brand image	0.06	0.02	0.01
H1c	Live attendance → Brand awareness	−0.01	0.03	0.74
	Live attendance → Congruence	0.11	0.03	0.001
H2a	Live streaming → Brand loyalty	−0.01	0.03	0.97
H2b	Live streaming → Brand image	−0.06	0.03	0.01
H2c	Live streaming → Brand awareness	−0.01	0.03	0.73
	Live streaming → Congruence	0.15	0.03	0.001
	Congruence → Brand loyalty	0.71	0.02	0.001
	Congruence → Brand image	0.74	0.02	0.001
	Congruence → Brand awareness	0.57	0.02	0.001
Indirect effect				
H3a	Live attendance → Congruence → Brand loyalty	0.08	0.02	0.04–0.11
H3b	Live attendance → Congruence → Brand image	0.07	0.02	0.03–0.11
H3c	Live attendance → Congruence → Brand awareness	0.04	0.01	0.02–0.06
H3d	Live streaming → Congruence → Brand loyalty	0.14	0.03	0.09–0.22
H3e	Live streaming → Congruence → Brand image	0.13	0.03	0.08–0.21
H3f	Live streaming → Congruence → Brand awareness	0.08	0.02	0.05–0.13

SE = Standard error. CI = Confidence interval.

## 5 Discussion

### 5.1 Theoretical implications

The findings of the present study contribute to the growing body of literature on eSport sponsorship by offering insights into the relationship between eSport consumption, congruence, and brand equity. While prior research has emphasized the rapid industrial growth and increasing corporate investment in eSport (Rogers et al., 2020), the present study provides empirical evidence on the differential impact of live attendance and live streaming on key dimensions of brand equity—brand loyalty, brand image, and brand awareness. Consistent with prior work in the sport management field, which posits sport consumption as a central driver of brand-related outcomes (Baek et al., 2020; Song et al., 2025), our findings indicate that eSport consumption—live attendance and live streaming—positively influences brand image. However, their direct effects on brand loyalty and brand awareness were not supported. The findings diverge from traditional sponsorship literature theory, which often suggests a direct and positive association between exposure to sponsored content and loyalty (Mazodier and Merunka, 2012; Sasmita and Suki, 2015). Our findings challenge the assumption that all forms of eSport consumption uniformly translate into higher brand loyalty and awareness, offering a more nuanced understanding of how different consumption modes shape brand-related outcomes.

A central contribution of the present study lies in identifying congruence as a key mediating mechanism linking eSport consumption to brand equity. The findings indicate that congruence serves as a crucial cognitive cue that enhances consumer processing and internalization of sponsorship messages. When brands are perceived to align naturally with eSport, this congruence facilitates the transfer of positive associations to the brand, supporting prior research that emphasizes the importance of sponsor-sport fit in enhancing sponsorship effectiveness (Mazodier and Merunka, 2012; Shu et al., 2015). This is because sponsors endemic to the gaming or eSport industry—such as hardware manufacturers or energy drink brands—are perceived as natural fits, enhancing their perceived legitimacy and influence on consumers’ attitudes and behaviors (Shu et al., 2015). These natural fits affirm existing schemas, fostering stronger brand loyalty, improving brand image, and increasing brand awareness (Mazodier and Merunka, 2012). In contrast, low congruence sponsors may disrupt these schemas, making their presence feel disjointed or irrelevant within the eSport context, thereby diminishing the effectiveness of the sponsorship message. These findings are consistent with those from traditional sport sponsorship literature, which has long established that congruence between the sponsor and the sport property increases attitude toward the sponsor and purchase intentions (Aguiló-Lemoine et al., 2020; Cornwell et al., 2024).

The unsupported direct effects between live attendance and live streaming on brand loyalty and brand awareness suggest that mere exposure through eSport consumption may be insufficient to foster deeper consumer commitment and recognition. This finding diverges from traditional sport sponsorship literature, which often posits a linear relationship between fan engagement and brand loyalty (Mazodier and Merunka, 2012). One plausible explanation is that the digital and highly competitive nature of the eSport ecosystem dilutes the impact of standard sponsorship strategies. Consumers in the eSport environment may require more immersive and personalized brand experiences to establish lasting loyalty. Additionally, the lack of direct influence on brand awareness implies that brand messages, while visible, may struggle to achieve cognitive salience without a strong perceived alignment with the eSport context.

Our findings advance the eSport sponsorship literature by clarifying the conditions under which sponsorship can effectively influence brand equity. Specifically, the mediating role of congruence suggests that successful sponsorship outcomes are contingent on the perceived fit between the sponsoring brand and eSport. This insight extends previous work by Rogers et al. (2020) and Flegr and Schmidt (2022), who emphasize the importance of alignment between corporate image and sponsorship objectives in driving brand equity outcomes. Furthermore, the study responds to calls for additional research on the economic and marketing implications of eSport sponsorship (Flegr and Schmidt, 2022), providing actionable insights for sponsors seeking to leverage this rapidly growing market.

Furthermore, the differential effects of live attendance and live streaming suggest that sponsorship strategies should be tailored to the unique characteristics of each consumption mode. While live attendance offers immersive brand experiences that enhance congruence and brand image, live streaming provides sustained brand visibility through digital platforms. However, neither form of engagement directly influences brand loyalty or awareness without the mediating effect of congruence. This insight emphasizes the need for sponsors to prioritize congruence-building strategies, such as interactive content, co-branded experiences, and authentic integration within the eSport ecosystem, to maximize their impact on brand equity.

In sum, the current study contributes to the sport management literature by offering a comprehensive analysis of how eSport consumption and perceived congruence interact to shape brand equity outcomes. We advance our understanding of eSport sponsorship by demonstrating the pivotal mediating role of congruence and highlight the importance of aligning sponsorship strategies with the specific consumption contexts of live attendance and live streaming.

### 5.2 Practical implications

The findings of the current study provide actionable insights for eSport practitioners and marketers seeking to enhance brand equity through sponsorships. Despite the non-significant direct effects of live attendance and live streaming on brand loyalty and brand awareness, the results highlight the critical role of congruence in fostering stronger brand equity outcomes. These insights offer practical guidance for brands to design more effective sponsorship strategies that align with the unique characteristics of the eSport environment.

First, eSport marketers should prioritize enhancing perceived congruence between their brand and the eSport ecosystem. Given the significant mediating role of congruence, brands should carefully align their sponsorship messages, visual branding, and engagement strategies with the values and experiences associated with eSport. For example, sponsors can strengthen congruence by emphasizing innovation, competitiveness, and digital-savviness, which are core attributes of the eSport audience. Interactive activations, such as branded in-game items, live-streamed fan experiences, and exclusive eSport content, can reinforce this alignment and deepen consumers perceived fit between the brand and the eSport environment.

Second, the findings suggest that digital platforms provide an effective avenue for brands to build brand image and awareness when congruence is effectively established. Since live streaming platforms such as Twitch and YouTube offer immersive, real-time interactions with fans, sponsors should leverage these platforms to deliver personalized and engaging experiences (Qian and Seifried, 2023). This can include integrating sponsor messages organically into the broadcast through product placements, co-branded content, and influencer collaborations. These activations should prioritize authenticity, as eSport audiences are highly attuned to inauthentic marketing efforts (Speed and Thompson, 2000). By doing so, sponsors can leverage real-time moments and fan interactions to create a compelling narrative that resonates with eSport enthusiasts.

Third, the findings emphasize the importance of tailoring sponsorship strategies to each consumption channel. For live attendance, sponsors should focus on creating experiential activations that foster emotional engagement and prolonged brand exposure. Physical installations, fan engagement zones, and exclusive merchandise can offer attendees tangible touchpoints that reinforce brand associations. In contrast, for live streaming, brands should focus on maintaining persistent visibility through on-screen branding, interactive chat features, and seamless product integrations. By aligning these activations with the eSport experience, brands can enhance the perception that they are an integral part of the community.

Finally, the present study offers important insights for non-endemic sponsors seeking to enter the rapidly expanding eSport market. The findings suggest that non-endemic brands can successfully enhance their brand image when perceived congruence is carefully managed. To achieve this, non-endemic sponsors should develop sponsorship programs that authentically connect their brand with the eSport culture and community. This involves crafting narratives that highlight shared values, such as teamwork, technological innovation, or excitement. By fostering congruence and tailoring activations to each consumption channel, non-endemic brands can effectively engage with the highly sought-after eSport audience and strengthen their market position (Huettermann et al., 2023).

### 5.3 Limitations and future research

In the present study, several limitations should be acknowledged, offering directions for future research. First, we delimited LoL as the eSport game. However, different eSport genres, such as first-person shooters (e.g., Overwatch), real-time strategy games (e.g., StarCraft II), and multiplayer online battle arenas (e.g., LoL), may present unique audience characteristics and engagement patterns (Kim et al., 2025). Future research should explore whether the effects of live attendance, live streaming, and congruence on brand equity differ across various eSport genres.

Second, the study’s findings are based on data collected from a specific cultural context (i.e., South Korea), which may limit the generalizability across different cultural and geographic regions (Ribeiro et al., 2023). Given the global nature of eSports, future research should adopt a cross-cultural comparative approach to investigate how cultural orientations influence eSport consumption behaviors, perceptions of congruence, and subsequent brand equity outcomes. Such research would provide a more comprehensive understanding of how sponsorship effectiveness varies in diverse cultural settings.

Third, the current study focused exclusively on professional eSports, leaving the strategic management and sponsorship dynamics of amateur eSports underexplored. Amateur eSports represent a growing segment with unique community-driven engagement that may offer additional avenues for brand activation (Butcher and Teah, 2023). Future research could examine how sponsorship strategies differ between professional and amateur eSports and assess whether the mechanisms driving perceived congruence and brand equity outcomes are consistent across these levels.

Finally, future studies could explore the role of emerging technologies and new sponsorship activation strategies within the eSport ecosystem. As virtual and augmented reality technologies become increasingly integrated into eSports experiences, these innovations may offer novel pathways for enhancing perceived congruence and fostering stronger brand connections (Kim et al., 2025). Investigating how these technological advancements shape consumer perceptions and brand outcomes represents an important avenue for future research.

## Data Availability

The raw data supporting the conclusions of this article will be made available by the authors, without undue reservation.

## References

[ref1] Abdolmaleki H. Pizzo A. D. Baker B. J. Mahmoudi A. Ghahfarokhi E. A. (2025). Esports in emerging markets: a balanced scorecard approach to LAN gaming centers in Iran. J. Glob. Sport Manag. 10, 297–317. doi: 10.1080/24704067.2023.2261444

[ref2] Aguiló-Lemoine À. E. Rejón-Guardia F. García-Sastre M. A. (2020). Congruence effects on the effectiveness of sponsorship of sport event websites: an experimental approach. Sustain. For. 12:8173. doi: 10.3390/su12198173

[ref3] Anderson J. R. (1983). The architecture of cognition: Harvard University Press.

[ref4] Baek W. Y. Kim K. Kim D. H. Byon K. K. (2020). The impacts of the perceived golf course brand globalness on customer loyalty through multidimensional perceived values. Sustain. For. 12:978. doi: 10.3390/su12030978

[ref5] Boronczyk F. Breuer C. (2021). The company you keep: brand image transfer in concurrent event sponsorship. J. Bus. Res. 124, 739–747. doi: 10.1016/j.jbusres.2019.03.022

[ref6] Brand L. Ströbel T. (2024). A victimless crime’? Implications of eSports extensions of sport club brands for brand management from a multi-actor-dominant logic. J. Glob. Sport Manag., 1–25. doi: 10.1080/24704067.2024.2438718, PMID: 40475060

[ref7] Bridges S. Keller K. L. Sood S. (2000). Communication strategies for brand extensions: enhancing perceived fit by establishing explanatory links. J. Advert. 29, 1–11. doi: 10.1080/00913367.2000.10673620

[ref8] Brislin R. W. (1970). Back-translation for cross-cultural research. J. Cross-Cult. Psychol. 1, 185–216. doi: 10.1177/135910457000100301

[ref9] Buchholz K. (2021). The world’s top 10 gaming markets. Available online at:https://www.statista.com/chart/25593/biggest-video-game-markets/

[ref10] Butcher L. Teah K. (2023). Setting the research agenda of the eSports revolution. J. Glob. Sport Manag. 8, 455–459. doi: 10.1080/24704067.2023.2201588

[ref11] Calapez A. Ribeiro T. Almeida V. Pedragosa V. (2024). Esports fan identity toward sponsor–sponsee relationship: an understanding of the role-based identity. Int. J. Sports Mark. Sponsorsh. 25, 42–66. doi: 10.1108/IJSMS-02-2023-0030

[ref12] Close Scheinbaum A. Lacey R. Drumwright M. (2019). Social responsibility and event-sponsor portfolio fit: positive outcomes for events and brand sponsors. Eur. J. Mark. 53, 138–163. doi: 10.1108/EJM-05-2018-0318

[ref13] Cornwell T. B. Frank A. Miller-Moudgil R. (2024). A research agenda at the intersection of sport sponsorship and service. J. Serv. Manag. 35, 108–126. doi: 10.1108/JOSM-02-2022-0057

[ref14] Cornwell T. B. Kwon Y. (2020). Sponsorship-linked marketing: research surpluses and shortages. J. Acad. Mark. Sci. 48, 607–629. doi: 10.1007/s11747-019-00654-w

[ref15] Davtyan D. Tashchian A. (2022). Exploring the impact of brand placement repetition on the effectiveness of umbrella branding. J. Prod. Brand. Manag. 31, 1077–1090. doi: 10.1108/JPBM-02-2021-3381

[ref16] Flegr S. Schmidt S. L. (2022). Strategic management in eSports–a systematic review of the literature. Sport Manage. Rev. 25, 631–655. doi: 10.1080/14413523.2021.1974222

[ref17] Fornell C. Larcker D. (1981). Evaluating structural equation models with unobservable variables and measurement error. J. Mark. Res. 18, 39–50. doi: 10.1177/002224378101800104

[ref18] Hair J. F. Black W. C. Babin B. J. Anderson R. E. (2010). Multivariate data analysis. Upper Saddle River, NJ: Prentice Hall.

[ref19] Huettermann M. Trail G. T. Pizzo A. D. Stallone V. (2023). Esports sponsorship: an empirical examination of esports consumers’ perceptions of non-endemic sponsors. J. Glob. Sport Manag. 8, 524–549. doi: 10.1080/24704067.2020.1846906

[ref20] Hutabarat P. Gayatri G. (2014). The influence of sponsor-event congruence in sponsorship of music festivals. South East Asian J. Manag. 8, 47–64. doi: 10.21002/seam.v8i1.3101

[ref21] Jang W. W. Kim K. A. Byon K. K. (2020). Social atmospherics, affective response, and behavioral intention associated with esports events. Front. Psychol. 11:1671. doi: 10.3389/fpsyg.2020.01671, PMID: 32849018 PMC7403203

[ref22] Keller K. L. (1993). Conceptualizing, measuring, and managing customer-based brand equity. J. Mark. 57, 1–22. doi: 10.1177/002224299305700101

[ref23] Kim K. A. Byon K. K. Baek W. Williams A. S. (2019). Examining structural relationships among sport service environments, excitement, consumer-to-consumer interaction, and consumer citizenship behaviors. Int. J. Hosp. Manag. 82, 318–325. doi: 10.1016/j.ijhm.2018.10.004

[ref24] Kim K. Jang W. Kim K. A. Hur C. H. (2025). “The intersection of technology, media, and sport. Conceptualizing digital sport” in Routledge handbook of sport communication. ed. Pedersen P. M.. 2nd ed (Oxon: Routledge), 352–364.

[ref25] Mael F. Ashforth B. E. (1992). Alumni and their alma mater: a partial test of the reformulated model of organizational identification. J. Organ. Behav. 13, 103–123. doi: 10.1002/job.4030130202

[ref26] Marcus C. (2023). Esports audience demographics 2023. Colormatics. Available online at:https://www.colormatics.com/article/esports-audience-demographics/

[ref27] Mazodier M. Merunka D. (2012). Achieving brand loyalty through sponsorship: the role of fit and self-congruity. J. Acad. Mark. Sci. 40, 807–820. doi: 10.1007/s11747-011-0285-y

[ref28] Meenaghan T. (1991). The role of sponsorship in the marketing communications mix. Int. J. Advert. 10, 35–47. doi: 10.1080/02650487.1991.11104432

[ref29] Meng-Lewis Y. Wong D. Liu C. (2024). The emergent community of esports fans in Japan: an analysis of motivations and preferences. Manag. Sport Leis., 1–32. doi: 10.1080/23750472.2024.2373149, PMID: 40475060

[ref30] Moharana T. R. Roy Bhattacharjee D. Pradhan D. Kuanr A. (2023). What drives sponsorship effectiveness? An examination of the roles of brand community identification, brand authenticity, and sponsor–club congruence. Psychol. Mark. 40, 1211–1236. doi: 10.1002/mar.21802

[ref31] Newzoo (2022) Newzoo’s global esports & live streaming market report 2022 Newzoo. Available online at:https://newzoo.com/resources/trend-reports/newzoo-global-esports-live-streaming-market-report-2022-free-version

[ref32] Nunnally J. C. Bernstein I. H. (1994). Psychometric theory. New York: McGraw Hill.

[ref33] Pizzo A. D. Su Y. Scholz T. Baker B. J. Hamari J. Ndanga L. (2022). Esports scholarship review: synthesis, contributions, and future research. J. Sport Manage. 36, 228–239. doi: 10.1123/jsm.2021-0228

[ref34] Pu H. Xiao S. Kota R. W. (2022). Virtual games meet physical playground: exploring and measuring motivations for live esports event attendance. Sport Soc. 25, 1886–1908. doi: 10.1080/17430437.2021.1890037

[ref35] Qian T. Y. Seifried C. (2023). Virtual interactions and sports viewing on social live streaming platforms: the role of co-creation experiences, platform involvement, and follow status. J. Bus. Res. 162:113884. doi: 10.1016/j.jbusres.2023.113884

[ref36] Ribeiro T. Almeida V. Calapez A. Matsuoka H. Yamashita R. (2023). Esports and Olympic games: a cross-cultural exploration of the player support behaviour towards the Olympics. Int. J. Sports Mark. Sponsor. 24, 700–721. doi: 10.1108/IJSMS-12-2022-0215, PMID: 35579975

[ref37] Rogers R. Farquhar L. Mummert J. (2020). Audience response to endemic and non-endemic sponsors of esports events. Int. J. Sports Mark. Sponsor. 21, 561–576. doi: 10.1108/IJSMS-09-2019-0107, PMID: 35579975

[ref38] Rumelhart D. E. (1980). “Schemata: the building blocks of cognition” in Theoretical issues in reading comprehension. eds. Spiro R. J. Bruce B. C. Brewer W. E. (Hillsdale, New Jersey: Lawrence Erlbaum Associates), 33–58.

[ref39] Ruth J. A. Simonin B. L. (2003). “Brought to you by Brand a and Brand B” investigating multiple sponsors’ influence on consumers' attitudes toward sponsored events. J. Advert. 32, 19–30. doi: 10.1080/00913367.2003.10639139

[ref40] Sasmita J. Suki N. M. (2015). Young consumers’ insights on brand equity: effects of brand association, brand loyalty, brand awareness, and brand image. Int. J. Retail Distrib. Manag. 43, 276–292. doi: 10.1108/IJRDM-02-2014-0024

[ref41] Shu S. T. King B. Chang C. H. (2015). Tourist perceptions of event–sponsor brand fit and sponsor brand attitude. J. Travel Tourism Mark. 32, 761–777. doi: 10.1080/10548408.2014.946576

[ref42] Sjöblom M. Macey J. Hamari J. (2020). Digital athletics in analogue stadiums: comparing gratifications for engagement between live attendance and online esports spectating. Internet Res. 30, 713–735. doi: 10.1108/INTR-07-2018-0304

[ref43] Song H. Kim K. A. Guo Y. Zhang J. J. (2025). Gameful experience and consumers’ intention to continue using running apps: the roles of brand attitude and negative online reviews. Asia Pac. J. Mark. Logist. 37, 1550–1571. doi: 10.1108/APJML-05-2024-0682

[ref44] Speed R. Thompson P. (2000). Determinants of sports sponsorship response. J. Acad. Mark. Sci. 28, 226–238. doi: 10.1177/0092070300282004

[ref45] Tajfel H. Turner J. (1979). “An integrative theory of intergroup conflict” in The social psychology of intergroup relations. eds. Austin W. Worchel S. (Monterey: Brooks/Cole Publishing Company), 33–47.

[ref46] Teal R. Roberts M. Harrigan P. Clarkson J. Rosenberg M. (2020). Leveraging spectator emotion: a review and conceptual framework for marketing health behaviors in elite sports. Sport Manage. Rev. 23, 183–199. doi: 10.1016/j.smr.2019.05.003

[ref47] Wohn D. Y. Freeman G. (2020). Live streaming, playing, and money spending behaviors in esports. Games Cult. 15, 73–88. doi: 10.1177/1555412019859184

[ref48] Woisetschläger D. M. Michaelis M. (2012). Sponsorship congruence and brand image: a pre-post event analysis. Eur. J. Mark. 46, 509–523. doi: 10.1108/03090561211202585

[ref49] Yoo B. Donthu N. (2001). Developing and validating a multidimensional consumer-based brand equity scale. J. Bus. Res. 52, 1–14. doi: 10.1016/S0148-2963(99)00098-3

[ref50] Zajonc R. B. (1968). Attitudinal effects of mere exposure. J. Pers. Soc. Psychol. 9, 1–27. doi: 10.1037/h00258485667435

